# A novel compound heterozygous *YY1AP1* variant in Grange syndrome: importance of early signs in preventing life-threatening vascular complications

**DOI:** 10.1038/s10038-026-01471-0

**Published:** 2026-03-26

**Authors:** Gul Unsel-Bolat, Neslihan Tezcan, Dilan Genç-Akdağ, Hamide Betül Gerik-Celebi, Alperen Tezcan, Hilmi Bolat

**Affiliations:** 1 Department of Child and Adolescent Psychiatry, Faculty of Medicine, Balikesir University, Balikesir, Türkiye; 2 Department of Medical Genetics, Faculty of Medicine, Balıkesir University, Balıkesir, Türkiye; 3 Department of Medical Genetics, Ataturk City Hospital, Balikesir, Türkiye; 4 Department of Radiology, Faculty of Medicine, Balıkesir University, Balıkesir, Türkiye

**Keywords:** Genetics, Psychiatric disorders

## Abstract

**Background:**

Grange syndrome is an ultra-rare autosomal recessive disorder caused by biallelic loss-of-function variants in the *YY1AP1* gene. It is clinically characterized by multisystem involvement, including vascular stenosis, brachysyndactyly, osteopenia, cardiac anomalies, and neurodevelopmental delay.

**Methods:**

Our case was followed up in the Child and Adolescent Psychiatry clinic with the diagnosis of intellectual disability. Whole-exome sequencing (WES) was performed, and Sanger sequencing was used to confirm the identified variant and conduct familial segregation analysis.

**Results:**

We report a 17-year-old Turkish female who presented with academic failure, speech delay, dysarthria, facial dysmorphism, and surgically corrected hand syndactyly. Notably, she exhibited no clinical signs of vascular stenosis or hypertension at the time of diagnosis. A novel compound heterozygous variant combination in *YY1AP1* (NM_139119.3) was identified: c.1489_1492del (p.Glu636ProfsTer13), previously reported as pathogenic, and c.1637_1638del (p.Pro546ArgfsTer26), a novel frameshift variant. These variants were maternally and paternally inherited, respectively.

**Conclusion:**

This is the first reported case of Grange syndrome diagnosed based on neurodevelopmental and dysmorphic findings before the onset of vascular or hypertensive symptoms. Our findings highlight the importance of considering *YY1AP1*-related pathology in the differential diagnosis of intellectual disability and syndactyly, even in the absence of vascular features. Early genetic diagnosis enables clinical surveillance for life-threatening complications associated with this syndrome.

## Introduction

Grange syndrome (GRNG; OMIM #602531) is an ultra-rare autosomal recessive disorder characterized by vasculopathy, particularly of the cerebral, renal, abdominal, and coronary arteries; skeletal anomalies; and brachydactyly, syndactyly, bone fragility, developmental delay, and learning disabilities. GRNG is caused by homozygous or compound heterozygous loss-of-function variants in the YY1-associated protein 1 (*YY1AP1*) gene (OMIM *607860), located on chromosome 1q22. Loss of *YY1AP1* has been shown to impair the differentiation and proliferation of smooth muscle cells, critical for vascular integrity [1].

Grange et al. (1998) initially described the syndrome in four related individuals with renal artery stenosis, hypertension, congenital heart anomalies, brachydactyly, syndactyly, and learning disabilities. Up to now, only 22 patients reported, with biallelic *YY1AP1* variants confirmed [1–12].

Here, we report a female patient with a compound heterozygous *YY1AP1* variant who was diagnosed with Grange syndrome based on neurodevelopmental findings rather than vascular complications. We also provide a comparative summary of previously reported cases of Grange syndrome.

## Materials and methods

### Patient evaluation

A 17-year-old female patient with developmental delay and intellectual disability was evaluated for a possible genetic etiology by a child psychiatrist and a clinical geneticist. Clinical findings, developmental history, pedigree, and physical examination were comprehensively reviewed, and informed consent was obtained from her legal guardians.

### Genetic testing

Genomic DNA was isolated from peripheral venous blood using the High Pure PCR Template Preparation Kit.

(Roche Diagnostics, Mannheim, Germany). Clinical exome sequencing was performed using the Human Comprehensive Exome Panel (Twist Bioscience, USA), which targets approximately 4900 clinically relevant genes. Sequencing was carried out on the DNBSEQ-G400 platform (MGI Tech, China) with 150 bp paired-end reads, achieving a mean depth of coverage of 80–100×.

Adapter sequences were removed during demultiplexing. The reads were aligned to the GRCh38 with BWAMEM (Li and Durbin, 2010). Post-alignment processing (sorting, duplicate marking, and base quality recalibration) was performed using GATK. Variants were carried out using GATK HaplotypeCaller and filtered based on read depth, strand bias, call quality, and other quality metrics.

### Variant interpretation

Variant analysis was performed using GenomizeSeq (version 6.13.1) with GRCh38. Candidate variants were visualized using Integrative Genomics Viewer (IGV), filtered using Human Phenotype Ontology (HPO) terms, and annotated using HGMD, ClinVar, LOVD, VarSome, and Ensembl Variant Effect Predictor. Rare variants (MAF < 1%) with predicted high impact or potential splicing effects were prioritized, especially if consistent with the patient’s phenotype.

## Results

**Fig. 1:**
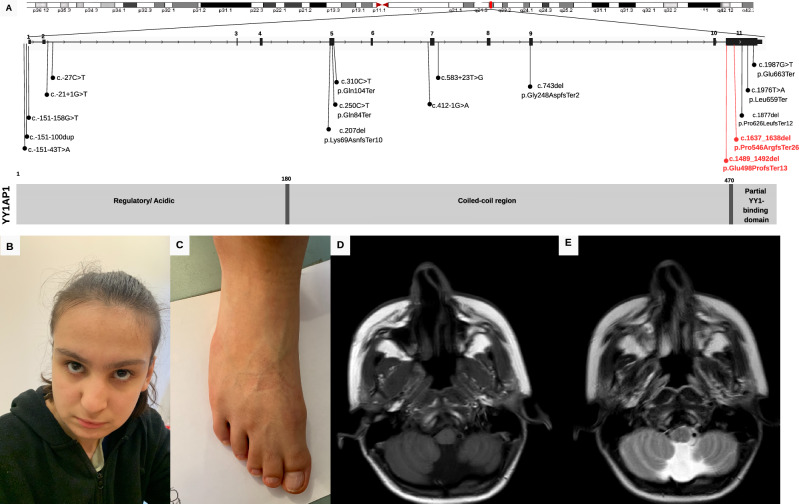
Schematic representation of YY1AP1 gene variants, clinical and neuroimaging findings of the patient. **A** Schematic overview of the YY1AP1 gene showing its chromosomal localization, exon–intron organization, and distribution of previously reported pathogenic variants. The upper panel depicts the chromosomal position, the middle panel illustrates the exon structure with corresponding cDNA and predicted protein changes, and the lower panel presents the functional domain organization of the YY1AP1 protein, including the regulatory/acidic region, coiled-coil region, and partial YY1-binding domain. **B** Facial appearance of the patient. **C** Clinical photograph demonstrating syndactyly. **D** Axial T1-weighted and **E** T2-weighted brain magnetic resonance images demonstrate an enlarged cisterna magna with cerebrospinal fluid signal characteristics (hypointense on T1-weighted and hyperintense on T2-weighted images), preserved cerebellar vermis, and normal fourth ventricle morphology, without evidence of mass effect or hydrocephalus, consistent with mega cisterna magna

The patient was born at term with a birth weight of 3000 g to healthy, non-consanguineous Turkish parents. On physical examination, dysmorphic facial features were noted, including low-set ears and a high-arched palate (Fig. 1). Also, she had dysarthria, bilateral shortening of the distal phalanges, pes planus, and accessory breast tissue along the bilateral sternocleidomastoid lines. Syndactyly between the third and fourth digits of both hands was surgically corrected at age four. At the time of evaluation, her weight was 42 kg (5th percentile), height was 160.2 cm (25th–50th percentile), and BMI was 16.37 kg/m². Neurodevelopmental assessment confirmed a diagnosis of mild intellectual disability. She was followed by the Child and Adolescent Psychiatry clinic with medical treatment and behavioral recommendations due to communication difficulties and social withdrawal.

Given these findings, genetic testing was performed and revealed a compound heterozygous variant in *YY1AP1* (NM_139119.3):

- c.1489_1492del (p.Glu636ProfsTer13) in exon 11, classified as pathogenic in ClinVar, inherited from the father.
- c.1637_1638del (p.Pro546ArgfsTer26), a novel frameshift variant in exon 11, not previously reported in ClinVar or GnomAD, inherited from the mother.

Subsequent evaluation focused on identifying other features of GRNG. Doppler ultrasonography of the bilateral renal, carotid, vertebral, and peripheral arteries revealed no stenosis, occlusion, or aneurysm. Echocardiography revealed a mitral valve prolapse. On T2-weighted brain magnetic resonance imaging, an enlarged cisterna magna demonstrating cerebrospinal fluid signal characteristics (hypointense on T1-weighted images and hyperintense on T2-weighted images) was observed. The cerebellar vermis and fourth ventricle morphology are normal. The findings in our patient are consistent with mega cisterna magna. (Fig. 1). No evidence of hypertension, bone fractures, abdominal artery stenosis, or ischemic cardiac events was detected at the time of diagnosis.

## Discussion

In this study, we reported a novel compound heterozygous variant combination —c.1489_1492del and c.1637_1638del—in the *YY1AP1* gene (NM_139119.3), providing novel insights into the phenotypic variability of GRNG syndrome presenting a case with a unique clinical trajectory.

**Fig. 2:**
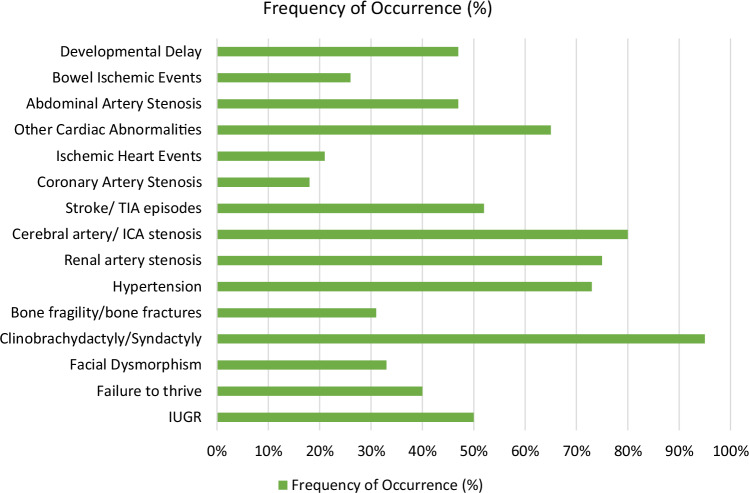
Frequency of clinical findings of Grange syndrome. ICA intra-cranial artery, IUGR intrauterine growth restriction, TIA transient ischemic attack

**Fig. 3:**
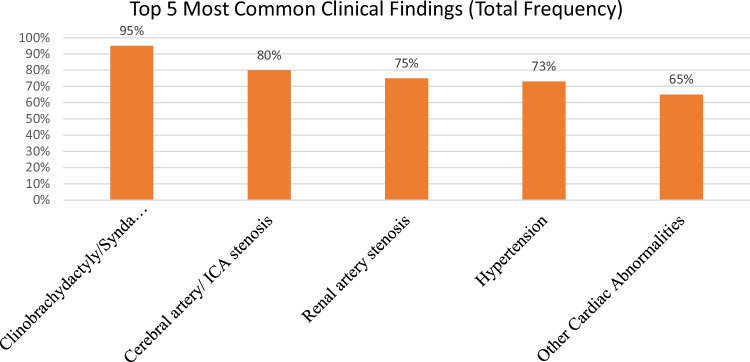
. Comparison of the 5 most common findings in patients with Grange syndrome. ICA intracranial artery

We compared the clinical features of our patient to those described in the literature, summarized in Table 1 and visualized in Fig. 2. Our patient exhibited the most frequently reported skeletal and neurodevelopmental features but lacked vascular pathology at the time of diagnosis, suggesting possible early-stage or incomplete expression of the syndrome. In our review, among the five most common clinical findings, syndactyly was observed in 95%, hypertension in %73, cerebral artery/ICA stenosis in 80%, renal artery stenosis in 75%, and other cardiac abnormalities in 65% (Fig. 3). These findings underscore the multisystemic nature of the condition and highlight its significant cardiovascular involvement. (Fig. 3)

**Table 1:** Comparison of the clinical features observed in our patient with those reported in the literature

Symptoms	Literature	Our patient	Total
Gender	16 females/7 males	Female	17 females/7 males
Age	0-53 years	17 years	0-53 years
IUGR	6/11 (54%)	No	6/12 (50%)
Failure to thrive	2/4 (50%)	No	2/5 (40%)
Facial Dysmorphism	4/14 (29%)	Yes	5/15 (33%)
Clinobrachydactyly/Syndactyly	19/22 (86%)	Yes	20/23 (95%)
Bone fragility/bone fractures	6/18(33%)	No	6/19 (31%)
Hypertension	16/21 (76%)	No	16/22 (73%)
Renal artery stenosis	15/19 (78%)	No	15/20 (75%)
Cerebral artery/ ICA stenosis	17/20 (85%)	No	17/21 (80%)
Stroke/ TIA episodes	10/18 (56%)	No	10/19 (52%)
Coronary Artery Stenosis	3/15 (20%)	No	3/16 (18%)
Ischemic Heart Events	4/18 (22%)	No	4/19 (21%)
Other Cardiac Abnormalities	11/17 (64%)	No	11/18 (65%)
Abdominal Artery Stenosis	9/17 (50%)	No	9/18 (47%)
Bowel Ischemic Events	5/18 (28%)	No	5/19 (26%)
Developmental Delay	10/22(45%)	Yes	11/23 (47%)

*ICA* intra-cranial artery, *IUGR* intrauterine growth restriction, *TIA* transient ischemic attack

Current literature indicates that vascular involvement in Grange syndrome shows marked variability in age of onset, with some individuals developing significant vascular complications during adolescence or adulthood, suggesting that vascular manifestations may exhibit age-dependent penetrance [1, 2, 5, 10, 11]. A review of previously published cases, summarized in Table 2, demonstrates marked variability in the age of onset of vascular manifestations. The earliest presentation reported by Karakaya (2022) was hypertension at 4 months of age, whereas in other individuals clinically significant vascular stenoses emerged during late childhood, adolescence, or adulthood [3]. In some patients, progressive vascular disease led to severe complications, including ischemic stroke, transient ischemic attack, or major cardiac events. These observations indicate that vascular involvement in GRNG does not follow a uniform timeline and may represent a dynamic and potentially progressive process. Therefore, we follow out patient for any signs of hypertension or vascular stenosis throughout life.

**Table 2:** Detailed clinical presentations of patients with Grange syndrome according to age periods

Case No.	Reference	Age (years)	Sex	0–6 Months	6–36 Months	3–6 Years	6–12 Years	12–18 Years	>18 Years
1	Grange et al. [2]	29	F	Born at term; PDA, VSD at 4 months Bicuspid aortic valve; systolic hypertension		Bone fracture (4)	Bone fracture (age 7–8)	LD	Cerebral artery stenosis (26); LV hypertrophy (26); renal vascular pruning; LAD stenosis; abdominal artery stenosis; ischemic heart events; bicuspid aortic valve; renal artery stenosis
2	Grange et al. [2]	27	M	Born at term		Bone fracture; HT	Behavior and learning problems during grade school	Graduated from high school after placement in a regular school	Abdominal artery stenosis, ischemic heart events, bicuspid aortic valve, renal artery stenosis
3	Grange et al. [2]	18	F	Born at term; bone fracture	Developmental delay at 7 months; renal USG: small kidney; HT (4 months); weight gain; renal artery stenosis (15 m); balloon angioplasty (28 m); bone fracture	Syndactyly corrected early	ICA occlusion (10); reading level: 4th grade	Renal artery stenosis (15); ischemic heart event → sudden death (18)	—
4	Grange et al. [2]	15	M	Born at term	Bone fracture (22–26 m); lacrimal duct blockage (22 m)	Bone fracture (4); HT (5); LV hypertrophy (6)		Chest pain (13); coronary artery narrowing; renal and abdominal artery stenosis (14); osteopenia; borderline ID	—
5	Weymann et al. [10]	15	M		HT (3); bone fracture (3)	Fracture (4); Right intraabdominal testis surgery (5); hernia repair (7)		ICA stenosis & basilar artery rupture (15); frontal ischemia; HT; LV hypertrophy; osteopenia; borderline ID	—
6	Wallerstein et al. [9]	3	F	Born at term; Apgar 9 at 1 and 5 min; HT (4 months)	Small kidney (diagnosed by renal USG at 6 m); delay in development (at 7 m); ventricular hypertrophy weight gain (at 15-20 m); renal artery stenosis (at 15 m); balloon angioplasty (at 28 m); bone fracture (at 22–26 m); blocked lacrimal duct (at 22 m)	Poor articulation			
7	Volonghi et al. [8]	18	F	—	Underwent surgical correction for syndactyly (at 1)	—	LD	Borderline ID; cerebral artery stenosis (18); renal, abdominal artery stenosis (18); HT; LV hypertrophy	—
8	Guo et al. [1]	<10	F	—	—	Borderline ID	—	—	—
9	Rath et al. [5]	25	F	Born at term	—	—	—	ICA stenosis (15); abdominal artery stenosis (15); renal artery stenosis (15); HT(15); well-performing university student	—
10	Rath et al. [5]	17	M	—	—	—	—	HT (17); ICA stenosis (15); bone fragility; LD	—
11	Rath et al. [5]	20	F	—	Syndactyly corrected at early childhood	—	—	Renal artery stenosis (13-16); ICA stenosis (13-16); HT	—
12	Alamo et al. [12]	53	F		—		—	Migraines (12)	HT (25); hypothyroidism (38); ischemic stroke (42); ICA & abdominal artery stenosis; renal artery stenosis (52); GRNG dx (51)
13	Saida et al. [6]	7	F	Oligohydramnios, IUGR, born at 32 w	Seizures, hemiparesis, thalamocaudate hemorrhage (15 m)	HT (3)	Genetic counseling (7); mild cognitive impairment; small left kidney	—	—
14	Raggio et al. [4]	7	M	Born at 36 w		HT (6); subclinical hypothyroidism; ↑LDL, TG; TIA (6)	ICA stenosis (7); renal artery stenosis (9)	—	—
15	Karakaya et al. [3]	1.5	F	Born at term PDA (diagnosed at 6 m)	HT(1); genetic counseling; subdural hematoma (at 15 m)	—	—	—	—
16	Viora et al. [7]	3	M	Born at 35 w	Ischemic stroke (at 16 m); cerebral atrophy (at 16 m)		—	—	—
17	Viora et al. [7]	9	F	Born at 37 weeks; IUGR; cardiomegaly	Developmental delay (walking at 18 months, language delay); ID	—	—		—
18	Viora et al. [7]	29	M		—	Renal artery stenosis (6)	—	Kidney failure (13); HT; multiple artery stenosis	—
19	Viora et al. [7]	0.6	—	IUGR; ventricular hypertrophy	—	—	—	—	—
20	Alsadiqi et al. [11]	14	F	—	—	—	Renal artery stenosis (10); HT (10) chronic watershed ischemic changes (10); TIA (11); ICA stenosis (11)	Celiac and superior mesenteric arteries (12)	
21	Alsadiqi et al. [11]	10	F	—	—	—	—	—	—
22	Alsadiqi et al. [11]	8	M	—	—	—	—	—	—
23	Our Patient	17	F		—	underwent syndactyly surgery in early childhood	—	ID, dysarthria	—

*PDA* patent ductus arteriosus, *VSD* ventricular septal defect, *HT* hypertension, *ID* intellectual disability, *LD* learning disability, *ICA* internal carotid artery, *MR* mental retardation, *IUGR* intrauterine growth restriction, *TIA* transient ischemic attack, *LV* left ventricular, *GRNG* gene-related neurovascular genetic condition

Neurodevelopmental features show additional heterogeneity. While some individuals demonstrate normal cognitive performance, others exhibit learning difficulties, borderline intellectual functioning, or overt intellectual disability. Although neurocognitive impairment may be exacerbated by cerebrovascular events, developmental delay has also been reported in certain cases without overt vascular pathology. In contrast, congenital anomalies are more frequently described in early life.

When considered in the context of previously reported cases, her presentation supports the concept of substantial phenotypic variability and suggests that neurodevelopmental manifestations may constitute an early or even predominant feature in a subset of patients. Our patient differs from most previously reported cases in that the diagnosis of Grange syndrome was established during the evaluation of neurodevelopmental delay and intellectual disability, in the absence of clinically evident vascular disease. In almost all previous reports, genetic testing was initiated due to symptoms of hypertension or vascular stenosis.

This unique presentation, in which our patient was diagnosed during the evaluation of neurodevelopmental delay and intellectual disability, despite the absence of clinically evident vascular disease, highlights the importance of early genetic diagnosis. Early genetic diagnosis may allow clinicians to proactively monitor patients for the development of vascular or hypertensive symptoms, which can manifest later in life and lead to significant morbidity and mortality. GRNG syndrome is rare, but its severity suggests that some cases may go undiagnosed, leading to fatal outcomes. Understanding the genetic basis of neurodevelopmental disorders not only supports diagnosis but also facilitates the early detection and management of emerging conditions associated with the syndrome.

The mechanisms underlying the marked variable expressivity of GRNG remain incompletely understood. Most reported pathogenic variants in *YY1AP1* are loss-of-function variants, predominantly nonsense or frameshift changes, distributed throughout the gene, with relative enrichment in the central and C-terminal regions (Fig. 1). A clear genotype–phenotype correlation has not yet been established. Nevertheless, differences in variant position and potential residual protein function may contribute to clinical heterogeneity.

In our patient, both frameshift variants (p.Glu498ProfsTer13 and p.Pro546ArgfsTer26) localize to the distal portion of the protein encoded by exon 11. These variants are predicted to truncate the C-terminal YY1interaction region while potentially preserving portions of the N-terminal and central coiled-coil domains. Although speculative, partial preservation of domains involved in vascular integrity could be associated with delayed or attenuated vascular manifestations, whereas disruption of the C-terminal regulatory region may contribute to the prominent neurodevelopmental phenotype observed in this case.

*YY1AP1* is predominantly expressed in muscle and testicular tissues; however, transcriptomic data demonstrate detectable expression in the brain, particularly within the cerebral hemispheres [1]. The protein interacts with the transcription factor YY1 and plays a key role in chromatin remodeling and transcriptional regulation processes essential for both vascular development and neuronal differentiation [1, 7]. Disruption of *YY1AP1* may therefore affect downstream gene expression programs relevant to neurodevelopment. Although intellectual disability has been reported in only a limited number of GRNG cases [1, 2, 5, 7, 9, 10], the cumulative evidence, together with our findings, supports a broader neurodevelopmental dimension of the syndrome.

Taken together, the available data support the view that Grange syndrome represents a dynamic and evolving disease spectrum. Even in adolescents without overt vascular findings, long-term cardiovascular and cerebrovascular surveillance remains essential. Larger longitudinal cohorts will be required to clarify potential genotype–phenotype correlations, the temporal evolution of vascular involvement, and the contribution of genetic or environmental modifiers.

## Conclusion

In summary, this report expands the phenotypic spectrum of Grange syndrome by describing the first known case diagnosed based on neurodevelopmental features before the onset of vascular symptoms. The identification of a novel compound heterozygous variant in *YY1AP1* supports the gene’s role in vascular and neurodevelopmental processes. Our findings highlight the importance of considering Grange syndrome in the differential diagnosis of intellectual disability associated with syndactyly or congenital anomalies and the value of early genetic testing in preventing fatal complications.

### Limitation

In this study, genetic analysis was performed using a clinical exome panel targeting approximately 4900 disease-associated genes. While the panel includes a broad range of genes implicated in neurodevelopmental disorders and intellectual disability, it does not encompass the entire exome. Therefore, variants located in genes not included in the panel or in non-coding regions may not have been detected. The complete list of genes covered by the panel is provided in Supplementary Table 1.

## Supplementary information

CES Gene List

## Data Availability

All data generated or analyzed during this study are included in this article. Further inquiries can be directed to the corresponding author.
